# Adoptive immunotherapy for AML with CD123-engager T cells

**DOI:** 10.1186/2051-1426-3-S2-P3

**Published:** 2015-11-04

**Authors:** Challice Bonifant, David Torres, Nicholos Joseph, Carolyn Arber Barth, Mireya Velasquez, Xiao-Tong Song, Michele Redell, Stephen Gottschalk

**Affiliations:** 1 Baylor College of Medicine, Center for Cell and Gene Therapy, Houston, TX, USA; 2 Baylor College of Medicine, Houston, TX, USA

## Background

T cell immunotherapy is one promising approach to improve outcomes for patients with AML; however, infused T cells do not redirect resident T cells to tumors. To overcome this, we have genetically modified T cells to produce a secretable, bispecific T cell engager (ENG-T cells). Consistent synthesis of engagers by T cells should be superior to the direct infusion of the recombinant bispecific antibody, because bispecific proteins typically have short half-lives and do not accumulate at tumor sites. The goal of this project was to generate and characterize T cells expressing CD123-specific engagers and evaluate the feasibility of using transgenic expression of CD20 in combination with rituximab as a suicide gene.

## Methods

CD123-ENG T cells were generated by transducing T cells with a retroviral vector encoding a CD123-specific T cell engager consisting of a scFv recognizing CD123 linked to a scFv recognizing CD3. The vector also contained mOrange or CD20 as a second transgene. Effector function of CD123-ENG T cells was evaluated *in vitro* and in a xenograft model. Toxicity of CD123-ENG T cells and the functionality of the suicide gene were evaluated *in vitro* and *in vivo*.

## Results

Mean transduction efficiency was 76% (49-95%), and CD123-ENG and CD20.CD123-ENG T cells recognized CD123+ cells (MV-4-11, MOLM-1, KG1a, K562-CD123) as judged by cytokine production and cytolytic activity. CD123-negative cells (K562) were not recognized by CD123-ENG T cells. Likewise, control ENG-T cells were not activated by CD123+ cells. Antigen-dependent recognition was confirmed with cytotoxicity assays. Since CD123 is expressed on normal hematopoietic progenitor cells (HPCs), we evaluated the ability of CD123-ENG T cells to recognize normal HPCs in colony formation assays. Only at high CD123-ENG to HPC ratios did we observe a decline in colony numbers. In contrast, toxicity to primary leukemia samples was apparent even at low CD123-ENG T cell:leukemia cell ratios, indicating that CD123+ AML cells can be targeted while preserving normal HPCs. *In vivo*, CD123-ENG T cells and CD20.CD123-ENG T cells had potent anti-tumor activity in the KG1a/NSG xenograft model resulting in a significant survival advantage of treated animals in comparison to mice that received Control-ENG T cells (p=0.002). Lastly, CD20.CD123-ENG T cells were efficiently eliminated by rituximab in the presence of complement in contrast to CD123-ENG T cells.

## Conclusions

We have generated CD123-ENG T cells that can direct bystander T cells to CD123+ AML in an antigen-specific manner. These CD123-ENG T cells have powerful anti-AML activity *in vivo* and when further engineered to also express CD20, are eliminated by rituximab. These cells may present a promising addition to currently available AML therapies.

